# Metabolite-Centered Evaluation of Plant-Based Substrates: Integrated Profiling of Short-Chain Fatty Acids (SCFAs) and Neuroactive Compounds with Potential Relevance to the Gut–Brain Axis

**DOI:** 10.3390/molecules31122073

**Published:** 2026-06-12

**Authors:** Mustafa Yıldız

**Affiliations:** Faculty of Engineering and Natural Sciences, Department of Food Engineering, Istanbul Sabahattin Zaim University, 34303 Istanbul, Türkiye; m.yildiz@izu.edu.tr

**Keywords:** short-chain fatty acids, neuroactive compounds, plant-based substrates, metabolite-centered framework, in vitro fermentation, gut–brain axis-associated pathways

## Abstract

This study presents an integrated metabolite-centered framework for the comparative evaluation of plant-based substrates through the simultaneous profiling of fermentation-associated short-chain fatty acids (SCFAs) and neuroactive compounds within a single in vitro experimental platform. Unlike conventional studies focusing on individual metabolite classes, the present approach combines in vitro gastrointestinal digestion with simplified bacterial fermentation to characterize substrate-dependent metabolic responses under controlled experimental conditions. Concurrent evaluation of SCFA production and neuroactive compound formation enabled multidimensional assessment of fermentation-associated metabolite profiles and their potential biochemical interrelationships. Significant differences (*p* < 0.05) were observed among substrates in both SCFA production and neuroactive compound formation. Hemp seed flour exhibited the highest acetate concentration (4.67 mg/100 g) and γ-aminobutyric acid (GABA) level (114.00 µg/g), whereas lentil and corn flour showed elevated propionate levels. Chickpea and bulgur produced the highest butyrate concentrations. Among neuroactive compounds, bulgur exhibited the highest dopamine and serotonin levels, while lentil demonstrated a more balanced metabolite profile. Correlation analysis suggested exploratory associations between SCFA production and neuroactive compound formation. A strong positive correlation between acetate and GABA (r = 0.89) indicated potential co-variation between carbohydrate fermentation and neuroactive metabolite formation, whereas divergent dopamine and serotonin patterns suggested substrate-dependent metabolic differences. Functional mapping further classified substrates into SCFA-oriented, neuroactive compound–dominant, and mixed metabolic profile groups. Collectively, these findings support a metabolite-centered framework for comparative assessment of plant-based substrates based on fermentation-associated metabolite profiles obtained under controlled in vitro conditions. Although the simplified two-strain fermentation model does not reproduce the complexity of the human colonic microbiota, the observed substrate-dependent metabolic differences may provide preliminary insights into biochemical outputs potentially relevant to gut–brain axis-associated pathways. Further studies employing complex microbial communities and in vivo validation are required to confirm the physiological relevance of these findings.

## 1. Introduction

The gut microbiota, representing a highly dynamic ecosystem of bacteria, viruses, fungi, and yeasts, is increasingly recognized as a central regulator of host metabolism, immune function, and neurophysiological processes [1]. In the human gastrointestinal system, indigestible dietary components are anaerobically fermented by the colon microbiota, leading to the production of short-chain fatty acids (SCFAs), including acetate, propionate, and butyrate. These metabolites play important roles in maintaining gut integrity, regulating energy metabolism, and modulating inflammatory responses. Furthermore, SCFAs have been shown to affect blood–brain barrier function and microglia activity, thus contributing to gut–brain axis signaling [2]. Dietary composition is a significant determinant of microbiota structure and metabolic output, directly shaping microbial metabolite production [3,4,5]. Cereals and legumes rich in fermentable fibers, resistant starch, and complex carbohydrates provide the necessary substrates for microbial fermentation. This fermentation is widely known to be associated with SCFA production and its beneficial effects on colon health and metabolic homeostasis [6,7]. In addition to SCFAs, the gut microbiota can produce neuroactive compounds such as GABA, dopamine, and serotonin, thus influencing host physiology through gut–brain axis interactions [8,9]. Plant-based foods such as chickpeas (*Cicer arietinum*), lentils (*Lens culinaris*), bulgur (*Triticum durum*), and maize (*Zea mays*) represent major sources of fermentable substrates. Their composition, rich in dietary fiber, protein, and bioactive compounds, provides fermentation substrates and potential precursors associated with neuroactive metabolite formation [10,11]. Emerging plant-based ingredients such as hemp seed (*Cannabis sativa*) and terebinth (*Pistacia terebinthus*) have also attracted increasing interest due to their nutritional and functional properties [12]. However, despite the growing interest in diet–microbiota interactions, studies evaluating microbiota-associated metabolites have largely focused on either short-chain fatty acid (SCFA) production or microbial composition separately. Consequently, the integrated assessment of SCFAs and neuroactive compounds within the same experimental framework remains relatively limited.

Although SCFA production and fermentation—associated neuroactive compound out pouts have been extensively investigated, their combined and substrate-dependent evaluation within a unified experimental framework is still insufficiently explored. Although fecal inoculum-based fermentation systems provide greater physiological relevance, simplified microbial models may serve as controlled and reproducible exploratory platforms for comparative evaluation of substrate-dependent metabolic responses under standardized in vitro conditions. Therefore, the present study employed a simplified two-strain bacterial fermentation model designed as an exploratory mechanistic screening platform rather than as a direct representation of the ecological and metabolic complexity of the human colonic microbiota.

## 2. Results

### 2.1. Short—Chain Fatty Acid Production Composition of Plant-Based Substrates

Significant differences in short-chain fatty acid (SCFA) production were observed among plant-based substrates (*p* < 0.05) (Table 1).

As shown in Table 1, lentil exhibited the highest propionate concentration. Lentil exhibited the highest propionate concentration (4.83 mg/100 g), followed by corn (4.52 mg/100 g). Hemp seed showed the highest acetate level (4.67 mg/100 g), while chickpea (0.252 mg/100 g) and bulgur (0.272 mg/100 g) demonstrated relatively higher butyrate concentrations compared to other substrates. Overall, SCFA production profiles varied depending on substrate type, indicating substrate-specific fermentation patterns.

### 2.2. Neuroactive Compound Profiles During In Vitro Fermentation

Neuroactive production also showed significant variation across substrates (*p* < 0.05) (Table 2).

Hemp flour exhibited the highest γ-aminobutyric acid (GABA) concentration (114.00 µg/g). Bulgur showed the highest dopamine (82.90 µg/g) and serotonin (21.37 µg/g) levels. Lentil presented relatively high levels of both GABA and dopamine, while chickpea, corn flour, and terebinth displayed moderate neurotransmitter production.

### 2.3. Comparative Analysis of SCFAs and Neuroactive Compounds

**Figure 1:**
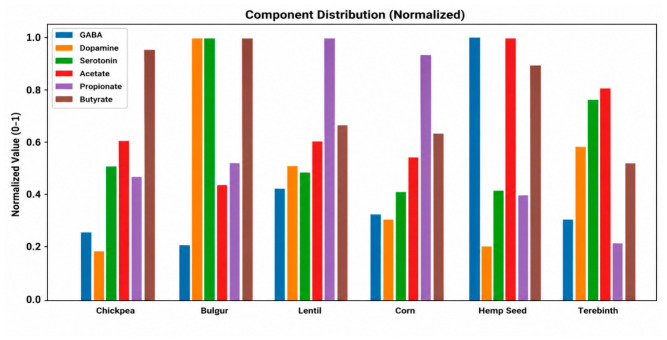
Normalized metabolite profiles (0–1 scale) of short-chain fatty acids and neurotransmitters across plant-based substrates. The normalization enables direct comparison of metabolite distributions and highlights substrate-specific metabolic signatures.

Normalized metabolite analysis (0–1 scale) revealed distinct clustering patterns among substrates (Figure 1).

Hemp was characterized by high GABA and acetate levels, while bulgur showed elevated dopamine and serotonin concentrations. Lentil exhibited a more balanced metabolite profile.

### 2.4. Correlation Analysis Between SCFAs and Neurotransmitters

To further explore the relationships between microbiota-derived metabolites, a correlation analysis was performed between short-chain fatty acids (SCFAs) and neuroactive compounds.

The Pearson correlation coefficients are presented in Table 3.

The values given in Table 3 represent the Pearson correlation coefficients (r) calculated among the plant-based substrates (*n* = 6). Because of the limited sample size, correlations are presented as exploratory descriptive relationships rather than statistically robust evidence of mechanistic relationships. Multiple test correction (Benjamini–Hochberg) was applied where appropriate.

**Figure 2:**
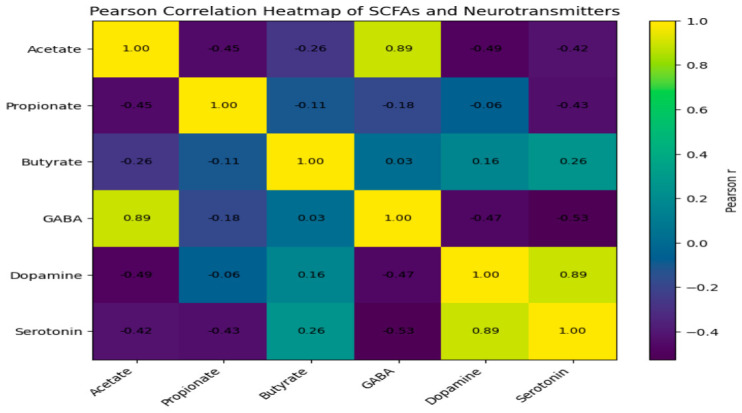
Pearson correlation heatmap showing relationships between SCFAs and neuroactive metabolites across plant-based substrates.

As shown in Figure 2, acetate exhibited a strong positive correlation with GABA (r = 0.89), while negative associations were observed with dopamine and serotonin. In contrast, propionate showed generally weak or negative correlations with neuroactive compounds, whereas butyrate displayed relatively limited positive associations, particularly with serotonin. These findings suggest potential metabolite-associated interactions under the applied in vitro fermentation conditions.

## 3. Discussion

### 3.1. Substrate-Dependent SCFA Production

The results clearly demonstrate that plant-based substrates generate distinct fermentation-associated short-chain fatty acid (SCFA) production profiles under the experimental conditions applied (Table 1). These variations likely reflect differences in substrate composition, including fermentable fiber structure, protein content, and bioactive precursor availability, which collectively influence bacterial metabolic responses [13,14,15]. Among the tested substrates, hemp seed exhibited the highest acetate production (4.67 mg/100 g), significantly exceeding all other samples. Acetate is known to enter systemic circulation and has been associated with multiple physiological processes, including blood–brain barrier transport and hypothalamic signaling [16,17,18]. Recent studies further suggest that diet-derived microbial acetate may contribute to microbiota–gut–brain axis-related metabolic communication through neuroimmune and neuroendocrine pathways [17,19]. This pronounced acetogenic profile suggests that hemp-derived substrates may differentially influence fermentation-associated metabolic outputs potentially relevant to metabolic and gut–brain axis-associated pathways.

In contrast, lentil (4.83 mg/100 g) and corn flour (4.52 mg/100 g) showed significantly elevated propionate production, indicating increased propionogenic metabolic activity under the experimental conditions applied. Propionate has been associated with metabolic regulation through its contribution to hepatic gluconeogenesis and gut hormone signaling [6,20]. Recent studies have further suggested that propionate may participate in appetite regulation, glucose homeostasis, and microbiota–host metabolic communication through gut-derived signaling pathways [18,21]. These findings suggest that lentil- and corn flour-based substrates may differentially influence fermentation-associated metabolic responses. Although butyrate concentrations were comparatively lower, chickpea (0.252 mg/100 g) and bulgur (0.272 mg/100 g) exhibited the highest levels among the tested substrates. Given the recognized role of butyrate in intestinal barrier integrity and inflammatory regulation [6,7], these substrates may be associated with fermentation patterns potentially relevant to intestinal epithelial health. In addition, recent evidence indicates that butyrate contributes to epithelial energy metabolism, tight junction maintenance, and microbiota–gut–brain axis-associated neuroimmune signaling [2,18].

Overall, SCFA production patterns were substrate-dependent and functionally differentiated under the experimental conditions applied, indicating that plant-based dietary matrices may influence fermentation-associated metabolic responses through differences in substrate composition. These findings support the concept that dietary composition is an important determinant of bacterial metabolic output profiles under controlled in vitro conditions and may provide preliminary insights into biochemical pathways potentially relevant to host metabolic and gut–brain axis-associated processes.

### 3.2. Neuroactive Compound Profiles

Neuroactive compound profiles further highlighted the substrate-dependent metabolic diversity of the evaluated plant-based samples (Table 2). Significant differences among substrates (*p* < 0.05) suggested that substrate composition may influence fermentation-associated neuroactive compound formation under the applied in vitro conditions. Among the tested samples, hemp seed flour exhibited exceptionally high GABA levels (114.00 µg/g), markedly exceeding those detected in the other substrates. Bulgur exhibited the highest dopamine (82.9 µg/g) and serotonin (21.37 µg/g) levels, indicating a pronounced neuroactive compound profile. These neuroactive compounds have been associated with neurophysiological processes in previous studies, suggesting that bulgur may generate distinct fermentation-associated neuroactive metabolite patterns under the experimental conditions applied [22,23]. Given that GABA has been associated with gut–brain signaling pathways in previous studies, this finding suggesting a distinct fermentation-associated GABA profile under the experimental conditions applied [9,23]. This effect may be attributed to the high protein content and amino acid composition of hemp, particularly its glutamate availability, which serves as a precursor for GABA-associated metabolic activity. The co-existence of high levels of short-chain fatty acids (SCFAs) and neuroactive compounds in hemp seeds may reflect substrate-dependent metabolic relationships under applied in vitro fermentation conditions. The high protein, carbohydrate, and amino acid composition of hemp seeds supports this hypothesis [12]. Lentils exhibited a balanced neurochemical profile, with relatively high levels of both GABA and dopamine. This balanced output likely reflects the combined presence of fermentable carbohydrates and protein-derived precursors, potentially contributing to diverse fermentation-associated metabolite patterns [13,24]. Pearson correlation analysis showed significant and substrate-dependent relationships between SCFA production and neuroactive compounds (Figure 2). Chickpea, corn flour, and Terebinth flour displayed moderate but distinct neurotransmitter production patterns, distinct fermentation-associated neuroactive metabolite profiles potentially relevant to gut–brain axis-associated pathways. These findings indicate that neuroactive compound profiles vary according to substrate composition and may be associated with distinct fermentation-related metabolic patterns under controlled in vitro conditions.

### 3.3. Correlation Analysis and Fermentation-Associated Metabolic Profile Interpretation

The integrated evaluation of short-chain fatty acids (SCFAs) and neuroactive compounds revealed substrate-dependent association patterns between fermentation-associated metabolite outputs. Pearson correlation analysis demonstrated distinct relationships between SCFA production and neuroactive compound formation under the applied in vitro fermentation conditions (Table 3; Figure 2). To the correlation analysis was based on a limited number of plant-based substrates (*n* = 6), the results were interpreted as exploratory indicators of co-variation rather than robust statistical evidence of mechanistic associations. Therefore, Pearson correlation coefficients (r) were presented primarily to provide a comparative overview of association trends under the applied in vitro conditions. Due to the limited sample size, *p* values and confidence intervals were not emphasized, as these estimates may be unstable and potentially misleading under low statistical power conditions.

The correlation analysis revealed that acetate showed a strong positive correlation with GABA (r = 0.89), suggesting a potential association between carbohydrate fermentation and neuroactive compound formation. As acetate is a major end-product of microbial carbohydrate metabolism, this relationship may reflect metabolic conditions favoring GABA formation through amino acid decarboxylation-related pathways. Similar associations between microbial fermentation products and neuroactive metabolite formation have previously been reported in studies investigating microbiota-associated metabolic activity and gut–brain axis-related pathways [18,22,25].

In contrast, acetate exhibited negative correlations with dopamine (r = −0.49) and serotonin (r = −0.42), suggesting that the formation of these compounds may involve different substrate utilization patterns and microbial metabolic activities, particularly those associated with aromatic amino acid metabolism [26,27]. Previous studies have suggested that microbial metabolism of tryptophan and tyrosine may influence neuroactive metabolite formation depending on substrate composition and microbial enzymatic activity [28,29].

Propionate showed weak and predominantly negative correlations with all neuroactive compounds, indicating a comparatively limited association with neuroactive metabolite formation under the applied experimental conditions. This observation may be associated with alternative propionate-producing pathways, including succinate and acrylate metabolism, which are more commonly linked to energy metabolism [7,28]. Butyrate displayed only a weak positive correlation with serotonin (r = 0.26), suggesting a potentially indirect association. Previous studies have reported that butyrate may influence host regulatory processes through mechanisms such as histone deacetylase (HDAC) inhibition and modulation of gene expression [30,31]. In addition, recent evidence has suggested that butyrate-mediated epigenetic modulation may be associated with neuroimmune signaling and intestinal barrier regulation through microbiota–gut–brain axis-related pathways [2,17,18].

Among the neuroactive compounds, dopamine and serotonin exhibited a strong positive correlation (r = 0.89), which may reflect partially overlapping precursor pathways involving aromatic amino acids such as tyrosine and tryptophan. Conversely, the negative correlations between GABA and both dopamine (r = −0.47) and serotonin (r = −0.53) may indicate substrate-dependent variations in microbial metabolic activity under the applied fermentation conditions.

Because the correlation analysis was based on a limited number of substrates (*n* = 6), the observed associations should be interpreted as exploratory indicators of co-variation rather than robust mechanistic evidence. Although several moderate-to-strong correlations were identified, the limited sample size reduces statistical power and increases uncertainty in correlation estimates. Therefore, the present findings should be considered preliminary and require validation using larger sample sets and more comprehensive microbial fermentation models.

**Figure 3:**
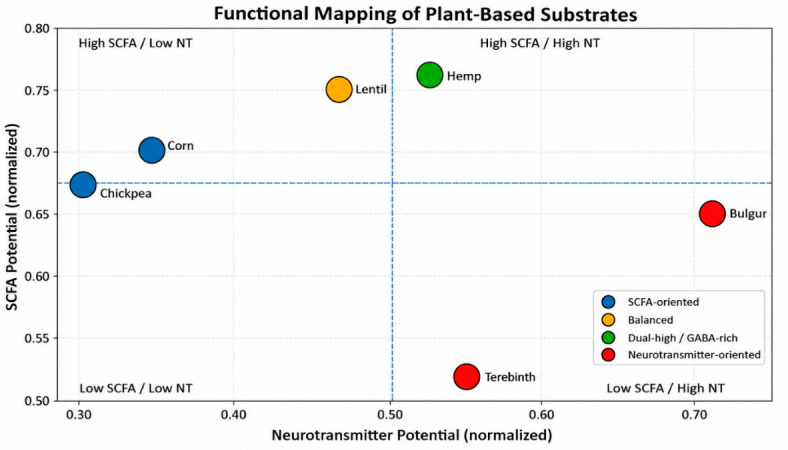
Neuroactive potential mapping of plant-based substrates based on combined neurotransmitter (dopamine + serotonin) and GABA production. Distinct clustering patterns indicate substrate-specific functional profiles related to gut–brain axis modulation.

Collectively, the observed SCFA and neuroactive compound profiles indicated that plant-based substrates generated distinct metabolite patterns under in vitro fermentation conditions. These substrate-dependent variations may provide a comparative framework for evaluating microbiota-associated metabolic responses and may be associated with the distribution trends observed in neuroactive potential mapping (Figure 3).

Based on the integrated metabolite profiles, substrates were comparatively grouped according to their dominant neuroactive characteristics (Table 4). Hemp seed flour exhibited relatively higher GABA-associated profiles, whereas bulgur and terebinth flour showed comparatively elevated dopamine and serotonin levels. Lentil displayed a more balanced neuroactive compound profile. Overall, these findings suggest that plant-based substrates may be associated with distinct fermentation-related metabolite patterns under controlled in vitro conditions.

As shown in Table 4. Importantly, this approach demonstrates that functional food properties can be systematically derived from microbiota-driven metabolite profiles.

### 3.4. Neuroactive Potential Mapping Interpretation

To further evaluate substrate-dependent metabolite patterns, a functional mapping approach integrating normalized SCFA and neuroactive compound potentials was applied (Figure 3). The resulting distribution revealed distinct clustering trends among plant-based substrates according to their relative metabolite profiles.

Hemp was positioned within the GABA-associated region, suggesting a potential relationship between amino acid-rich substrates and neuroactive metabolite formation. This observation is generally consistent with previous studies reporting that protein- and amino acid-containing substrates may support GABA-related metabolic pathways through microbial glutamate metabolism [18,22]. In contrast, bulgur and terebinth were located closer to the neurotransmitter-associated region, indicating potential associations with aromatic amino acid-derived metabolites such as dopamine and serotonin. These findings are consistent with previous reports suggesting that microbial tryptophan and tyrosine metabolism may contribute to neuroactive compound formation within gut-related metabolic pathways [27,28].

Lentil occupied an intermediate position, reflecting a comparatively balanced metabolite profile associated with both SCFA production and neuroactive compound formation. Such patterns may be related to the simultaneous presence of fermentable carbohydrates and amino acid precursors within the substrate matrix [7]. Chickpea and corn flour, on the other hand, were positioned within regions characterized by moderate SCFA production and comparatively lower neuroactive metabolite levels. To evaluate substrate-dependent metabolite patterns in greater detail, a functional mapping approach integrating normalized SCFA and neuroactive compound potentials was applied (Figure 3). The resulting spatial distribution revealed distinct clustering trends among plant-based substrates according to their relative metabolite profiles, suggesting differences in microbiota-associated metabolic responses under in vitro fermentation conditions. Hemp seed flour was positioned closer to the GABA-associated region, whereas bulgur and terebinth were located nearer to the neurotransmitter-associated region. Lentil occupied an intermediate position, indicating a comparatively balanced metabolite profile.

Collectively, these distribution patterns suggest that plant-based substrates may differentially influence microbiota-associated metabolic responses under in vitro fermentation conditions. Rather than generating uniform metabolite outputs, the tested substrates exhibited distinct SCFA and neuroactive compound profiles potentially associated with substrate composition and fermentation characteristics [29].

### 3.5. Metabolite-Centered Interpretation

**Figure 4:**
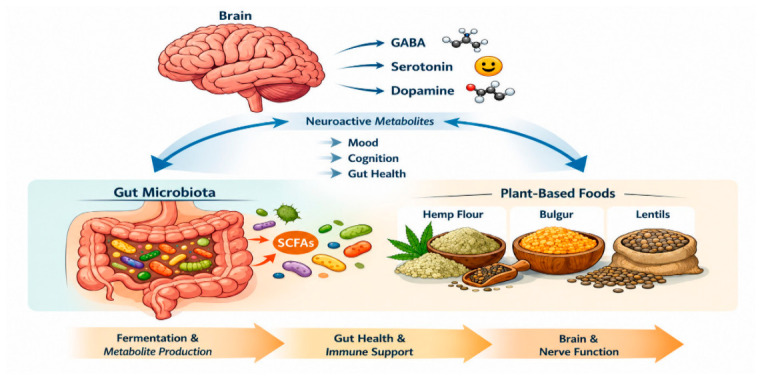
Conceptual framework illustrating fermentation-associated metabolite profiles generated from plant-based substrates under in vitro conditions. The diagram integrates substrate composition, simplified bacterial fermentation, and the formation of short-chain fatty acids and neuroactive compounds with potential relevance to gut–brain axis-associated pathways.

A critical consideration in interpreting microbiota-associated metabolite production is the distinction between pre-existing compounds and metabolites potentially produced during fermentation. While baseline metabolite concentrations (t_0_) and control fermentations were not directly assessed in this study, the observed post-fermentation metabolite patterns are generally consistent with previous reports showing relatively low baseline levels in crude plant substrates [9,22]. To provide a conceptual interpretation of the findings, the interactions between plant-based substrates, microbiota-associated metabolites, and gut-related metabolic pathways are schematically shown in Figure 4. The proposed framework suggests that substrate composition can influence microbial metabolic responses and lead to different SCFA and neuroactive compound profiles under in vitro fermentation conditions [22].

### 3.6. Methodological Considerations and Limitations

A limitation of the present study is the absence of direct baseline (t_0_) metabolite measurements and comprehensive control fermentations. Therefore, the detected SCFA and neuroactive compound levels should be interpreted as fermentation-associated metabolite profiles observed under standardized in vitro conditions rather than definitive evidence of de novo microbial metabolite biosynthesis. It is possible that a proportion of the detected metabolites originated from the substrates, fermentation medium, bacterial inoculum, or analytical background. However, because all substrates were evaluated using the same fermentation system and analytical workflow, the observed differences may still provide comparative insight into substrate-dependent metabolite patterns. Previous studies have reported relatively low baseline levels of SCFAs and microbiota-associated neuroactive compounds in raw plant substrates prior to fermentation, supporting the interpretation that microbial fermentation may contribute substantially to the observed metabolite profiles [2,9,18]. Future studies incorporating baseline measurements, controlled fermentation systems, and additional control groups will be necessary to more clearly distinguish substrate-derived and fermentation-associated metabolite contributions.

In addition, the present fermentation model represents a highly simplified experimental system and does not reproduce the ecological diversity, metabolic cross-feeding interactions, strict anaerobic conditions, or functional complexity of the human colonic microbiota. The two-strain bacterial system employing *Escherichia coli* and *Lacticaseibacillus casei* was intentionally designed as a controlled exploratory platform for comparative evaluation of substrate-dependent metabolic responses under standardized in vitro conditions rather than as a physiologically representative simulation of the intestinal microbiome. Therefore, the observed metabolite profiles should be interpreted as exploratory fermentation-associated metabolic outputs rather than direct representations of in vivo microbiota metabolism. Future studies employing fecal inoculum-based anaerobic fermentation systems, defined multi-species microbial consortia, and in vivo validation approaches are required to further evaluate the physiological relevance of the observed metabolite patterns.

## 4. Materials and Methods

### 4.1. Samples

Plant-based substrates, including chickpea (*Cicer arietinum* L.), bulgur (derived from *Triticum aestivum* L.), lentil (*Lens culinaris*), corn flour (*Zea mays* L.), hemp seed (*Cannabis sativa* L.), and terebinth (*Pistacia terebinthus* L.), were obtained simultaneously from a local market in İstanbul, Türkiye. The study included six plant-based product groups selected based on their common consumption and compositional diversity. To improve sample representativeness and minimize potential batch-related variability, three independent samples were collected for each product group. All samples were prepared according to the corresponding analytical protocols and analyzed separately using HPLC-based methods. Analytical measurements for all samples were performed in triplicate (*n* = 3) to ensure reproducibility and analytical reliability.

### 4.2. Chemical Reagents and Enzymes

All chemicals, enzymes, and analytical standards used in the present study were of analytical grade and obtained from Sigma-Aldrich (St. Louis, MO, USA). For the in vitro digestion procedure, α-amylase (1.5 U/mg), lipase (100–500 U/mg), pancreatin (8 × USP), pepsin (≥250 U/mg), bile salts mixture, mucin, bovine serum albumin, urea, uric acid, NaCl, KCl, CaCl_2_·2H_2_O, NaHCO_3_, hydrochloric acid (HCl), and sodium hydroxide (NaOH) were used. For SCFA analysis, analytical standards of acetic, propionic, and butyric acids, together with sodium dihydrogen phosphate (NaH_2_PO_4_), were used for chromatographic calibration and quantification.

### 4.3. Sample Preparation

Samples (5 g) were weighed into 50 mL Falcon tubes, diluted with ultrapure water to the desired volume, and centrifuged at 8000 rpm for 5 min. Pre-digestion samples were filtered through a 0.45 μm CA filter and transferred to vials prior to undergoing the in vitro digestion procedure.

### 4.4. Oral Phase

For the oral simulation, 5 mL of the prepared salivary solution was added to the weighed samples in a 100 mL Erlenmeyer flask. The mixture was homogenized and incubated at 37 °C in a shaking water bath for 5 min.

### 4.5. Gastric Phase

To simulate gastric digestion, 12 mL of gastric solution was added to the oral-phase mixture, which was then incubated at 37 °C for 2 h in a shaking water bath.

### 4.6. Intestinal Phase

Following the gastric phase, 10 mL of small intestinal solution and 5 mL of bile solution were added to the mixture. The resulting solution was incubated at 37 °C for 2 h in a shaking water bath. After incubation, the mixture was adjusted to the final volume, centrifuged, and filtered prior to downstream analysis.

### 4.7. In Vitro Model Design

**Figure 5:**
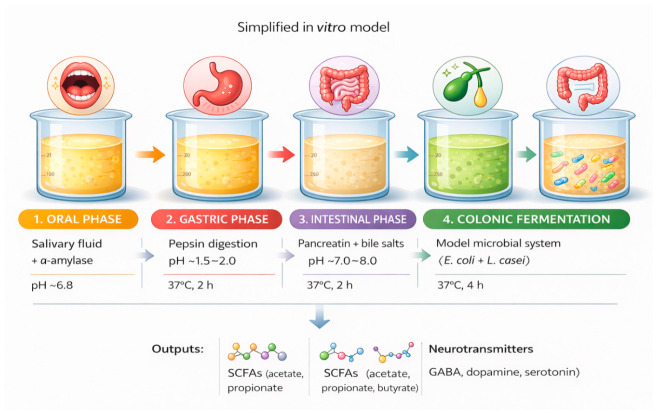
Schematic representation of the in vitro gastrointestinal digestion and subsequent colonic fermentation system based on the standardized INFOGEST protocol. The model includes oral, gastric, and intestinal digestion phases under controlled experimental conditions. Following digestion, the resulting digesta was subjected to in vitro colonic fermentation for the evaluation of short-chain fatty acids and neuroactive compounds [34,35].

The simplified bacterial fermentation model employing *Escherichia coli* and *Lacticaseibacillus casei* was intentionally designed as a controlled and reproducible exploratory screening system to evaluate substrate-dependent bacterial metabolic responses under standardized in vitro conditions. Although the selected microorganisms do not represent the ecological diversity, anaerobic dominance, interspecies interactions, or metabolic complexity of the human colonic microbiota, this reductionist approach enables comparative assessment of fermentation-associated metabolite profiles across different plant-based substrates under identical experimental conditions. Therefore, the present model should be interpreted as an exploratory in vitro fermentation platform rather than a direct representation of in vivo intestinal conditions. In vitro gastrointestinal digestion was performed using a standardized static digestion model based on the INFOGEST protocol. The model mimics the oral, gastric, and intestinal stages under physiologically determined conditions (Figure 5).

### 4.8. In Vitro Colonic Fermentation and HPLC Analysis of SCFAs and Neuroactive Compounds

Following in vitro gastrointestinal digestion, the undigested fraction was subjected to colonic fermentation to evaluate microbiota-associated metabolite production. The digestion residue was mixed with fermentation medium and incubated at 37 °C for 4 h under controlled laboratory conditions. To establish a simplified microbial fermentation model, cultures of *Escherichia coli* and *Lacticaseibacillus casei* were used as representative microorganisms. After fermentation, samples were centrifuged at 10,000× *g* for 10 min at 4 °C, and the supernatants were filtered through 0.22 µm membrane filters prior to chromatographic analysis. The study included three biological replicates per substrate, while each analytical determination was performed as three technical replicates.

Short-chain fatty acids (SCFAs; acetic, propionic, and butyric acids) were quantified using high-performance liquid chromatography (HPLC) according to the method described by De Baere [36]. Analyses were performed using a Shimadzu LC-20AD HPLC system equipped with a UV/PDA detector (Nakagyo-ku, Japan). Chromatographic separation was achieved using an ACE 5 C18 column (250 mm × 4.6 mm, 5 µm particle size) maintained at 30 °C. The mobile phase consisted of 0.1% phosphoric acid (Phase A) and methanol (Phase B) under gradient elution conditions at a flow rate of 1.0 mL/min. Detection was carried out at 210–220 nm. External calibration curves were prepared using analytical standards of acetic, propionic, butyric, and isobutyric acids at concentrations of 10, 25, 50, 100, and 200 ppm. SCFA concentrations were quantified based on retention times and calibration curves and expressed as mg/100 g sample.

Neuroactive compounds, including γ-aminobutyric acid (GABA), dopamine, and serotonin, were analyzed using an HPLC system (Shimadzu Nexera-i LC-2040C, Japan) equipped with a fluorescence detector (FLD) and an ACE 5 C18 column (250 × 4.6 mm, 5 µm particle size). Prior to analysis, samples were centrifuged at 10,000× *g* for 10 min at 4 °C and filtered through 0.22 µm syringe filters. Pre-column derivatization with o-phthalaldehyde (OPA) was applied for GABA determination. Chromatographic separation was performed using gradient elution with solvent A (phosphate/sodium acetate buffer) and solvent B (acetonitrile or methanol) at a flow rate of 0.8–1.0 mL/min, while the column temperature was maintained at 30 °C. Fluorescence detection was performed at excitation/emission wavelengths of 340/450 nm for OPA-derivatized GABA. Dopamine and serotonin were identified and quantified using corresponding external analytical standards under the same chromatographic conditions. Quantification was carried out using external calibration curves, and results were expressed as µg/g sample.

### 4.9. Statistical Analysis

Data were analyzed using one-way analysis of variance (ANOVA) to determine significant differences among substrates. Post hoc comparisons were performed using Tukey’s HSD test at a significance level of *p* < 0.05. All analyses were conducted using SPSS software (version 26.0; IBM Corp., Armonk, NY, USA).

## 5. Conclusions

This study demonstrated that plant-based substrates generate distinct profiles of short-chain fatty acids (SCFAs) and neuroactive compounds under controlled in vitro fermentation conditions. The findings suggested that substrate composition may influence microbiota-associated metabolite formation, resulting in variable SCFA and neuroactive compound patterns among the tested samples. In particular, acetate exhibited a strong positive association with GABA, whereas dopamine and serotonin showed different correlation patterns, indicating potential substrate-dependent variations in microbial metabolic responses under the applied experimental conditions. The combined evaluation of SCFAs and neuroactive compounds provided an exploratory metabolite-centered framework for comparative interpretation of fermentation-associated metabolic responses and highlighted potential relationships between dietary substrates and metabolite profile distributions. In addition, the neuroactive potential mapping approach revealed distinct distribution trends among plant-based substrates according to their dominant metabolite characteristics.

However, the present findings should be interpreted as preliminary metabolite-level observations obtained from a simplified two-strain mechanistic fermentation model rather than definitive evidence of physiological gut microbiota activity or direct gut–brain axis modulation. The experimental system was intentionally designed as a controlled exploratory in vitro platform for comparative assessment of substrate-dependent metabolite generation under standardized conditions and does not reproduce the ecological diversity, metabolic cross-feeding interactions, strict anaerobic conditions, or functional complexity of the human colonic microbiota. Furthermore, the limited substrate number, short fermentation duration, absence of microbiome characterization, and lack of in vivo validation restrict the broader biological interpretation of the observed associations. Therefore, future studies employing fecal inoculum-based anaerobic fermentation systems, defined multi-species microbial consortia, microbiome-integrated analyses, host-interaction models, and in vivo validation approaches are required to further evaluate the physiological relevance and translational applicability of the observed metabolite patterns.

## Figures and Tables

**Table 1:** Short-chain fatty acid concentrations in plant-based substrates after in vitro digestion and colonic fermentation (mean ± SD, *n* = 3; mg/100 g).

Sample	Acetate	Propionate	Butyrate
Chickpea	2.55 ± 0.05 ^c^	2.30 ± 0.10 ^c^	0.252 ± 0.008 ^a^
Bulgur	1.93 ± 0.13 ^d^	2.45 ± 0.05 ^c^	0.272 ± 0.013 ^a^
Lentil	2.80 ± 0.05 ^c^	4.83 ± 0.08 ^a^	0.188 ± 0.008 ^c^
Corn	2.45 ± 0.05 ^c^	4.52 ± 0.14 ^b^	0.168 ± 0.008 ^c^
Hemp seed	4.67 ± 0.16 ^a^	1.77 ± 0.08 ^d^	0.227 ± 0.015 ^b^
Terebinth	3.50 ± 0.30 ^b^	0.98 ± 0.08 ^e^	0.136 ± 0.006 ^d^

**Notes:** Values are expressed as mean ± standard deviation (*n* = 3). Different superscript letters within the same column indicate significant differences (*p* < 0.05) according to ANOVA followed by Tukey HSD test.

**Table 2:** Neuroactive compound concentrations in plant-based substrates after in vitro colonic fermentation (mean ± SD, *n* = 3; µg/g).

Sample	GABA (µg/g)	Dopamine (µg/g)	Serotonin (µg/g)
Chickpea	29.23 ± 0.87 ^e^	12.30 ± 0.25 ^f^	11.33 ± 0.86 ^c^
Bulgur	22.98 ± 0.56 ^f^	82.90 ± 2.58 ^a^	21.37 ± 1.06 ^a^
Lentil	47.80 ± 0.61 ^b^	41.97 ± 1.51 ^c^	10.78 ± 0.63 ^c^
Corn flour	36.40 ± 0.86 ^c^	24.93 ± 0.74 ^d^	8.37 ± 0.25 ^d^
Hemp flour	114.00 ± 0.87 ^a^	15.97 ± 0.49 ^e^	8.78 ± 0.18 ^d^
Terebinth flour	35.10 ± 0.56 ^d^	47.30 ± 1.07 ^b^	16.68 ± 0.28 ^b^

**Notes:** Values are expressed as mean ± standard deviation (*n* = 3). Different superscript letters within the same column indicate significant differences (*p* < 0.05) according to ANOVA followed by Tukey HSD test.

**Table 3:** Correlations between short-chain fatty acids and neuroactive metabolites in plant-based substrates.

GABA		Dopamine	Serotonin
Acetate	0.89	−0.49	−0.42
Propionate	−0.18	−0.06	−0.43
Butyrate	0.03	0.16	0.26

**Table 4:** Classification of plant-based substrates according to fermentation-associated SCFA and neuroactive compound profiles.

Substrate	Functional Properties Observed
Hemp flour	Highest GABA production → potential stress-reducing and neuroprotective effects [9,16]
Bulgur	Highest dopamine and serotonin levels → support for cognitive function and mood regulation [22,32]
Lentil	Balanced neurotransmitter production → overall support for the gut–brain axis [13,14]
Terebinth flour	Elevated dopamine and serotonin → high potential for neuroactive metabolite production [28,29,33]
Corn flour	Moderate GABA and dopamine levels → supportive functional effects [14,15]
Chickpea	Moderate serotonin and GABA levels → supportive role in gut health [24,32]

## Data Availability

The data presented in this study are available within the article.
